# ETV4 promotes late development of prostatic intraepithelial neoplasia and cell proliferation through direct and p53-mediated downregulation of p21

**DOI:** 10.1186/s13045-020-00943-w

**Published:** 2020-08-13

**Authors:** Irene Cosi, Annamaria Pellecchia, Emanuele De Lorenzo, Eugenio Torre, Michela Sica, Gabriella Nesi, Rosario Notaro, Maria De Angioletti

**Affiliations:** 1 Laboratory of Cancer Genetics, Core Research Laboratory, Istituto per lo Studio, la Prevenzione e la Rete Oncologica (ISPRO), Florence, 50139 Italy; 2 Doctorate School GenOMeC, University of Siena, Siena, Italy; 3 Department of Experimental and Clinical Biomedical Sciences, Section of Experimental Pathology and Oncology, University of Florence, 50134 Florence, Italy; 4 Division of Pathology, Department of Health Sciences, University of Florence, 50139 Florence, Italy; 5 ICCOM-National Council of Research, Sesto Fiorentino, Florence, 50019 Italy

**Keywords:** Prostate cancer, Mouse model, ETV4, ETS proteins, Cell proliferation, Cell cycle, p53, p21

## Abstract

**Background:**

ETV4 is one of the ETS proteins overexpressed in prostate cancer (PC) as a result of recurrent chromosomal translocations. In human prostate cell lines, ETV4 promotes migration, invasion, and proliferation; however, its role in PC has been unclear. In this study, we have explored the effects of ETV4 expression in the prostate in a novel transgenic mouse model.

**Methods:**

We have created a mouse model with prostate-specific expression of ETV4 (ETV4 mice). By histochemical and molecular analysis, we have investigated in these engineered mice the expression of p21, p27, and p53. The implications of our in vivo findings have been further investigated in human cells lines by chromatin-immunoprecipitation (ChIP) and luciferase assays.

**Results:**

ETV4 mice, from two independent transgenic lines, have increased cell proliferation in their prostate and two-thirds of them, by the age of 10 months, developed mouse prostatic intraepithelial neoplasia (mPIN). In these mice, *cdkn1a* and its p21 protein product were reduced compared to controls; p27 protein was also reduced. By ChIP assay in human prostate cell lines, we show that ETV4 binds to a specific site (-704/-696 bp upstream of the transcription start) in the *CDKN1A* promoter that was proven, by luciferase assay, to be functionally competent. ETV4 further controls *CDKN1A* expression by downregulating p53 protein: this reduction of p53 was confirmed in vivo in ETV4 mice.

**Conclusions:**

ETV4 overexpression results in the development of mPIN but not in progression to cancer. ETV4 increases prostate cell proliferation through multiple mechanisms, including downregulation of *CDKN1A* and its p21 protein product: this in turn is mediated through direct binding of ETV4 to the *CDKN1A* promoter and through the ETV4-mediated decrease of p53. This multi-faceted role of ETV4 in prostate cancer makes it a potential target for novel therapeutic approaches that could be explored in this ETV4 transgenic model.

## Background

Prostate cancer is a localized and indolent disease that becomes aggressive only in a small proportion of patients. Despite early diagnosis and the improved effectiveness of treatments, this cancer is the second leading cause of cancer death in males because of its high prevalence in elderly male population [1].

In almost half of patients with prostate cancer, the tumor carries one of recurrent translocations that place one of the genes from the ETS family (*ERG, ETV1, ETV4, ETV5, FLI1*) downstream to the promoter of a gene active in the prostate, with consequent aberrant overexpression of the respective *ETS* gene [2–5]. The role of the *ETS* genes in prostate carcinogenesis has been investigated in transgenic mice models with a prostate-specific ETS overexpression [6, 7]. The results have not been always concordant: some studies suggest that ERG or ETV1 overexpression promotes pre-malignant in situ lesions (equivalent to prostatic intraepithelial neoplasia, PIN) [8–12], whereas other studies suggest that this overexpression is not sufficient to cause the onset of cancer [13–18]. These variable results may be related to many factors such as transgene expression levels, transgene integration site, transgene structure, and what promoter drives transgene expression. The genetic background of mice and the timing of the analysis may also play a role, as in the case of human patients.

ETV4 is overexpressed in several cancers [19–24] and in a relatively small fraction of prostate cancers [25–29]. In vitro studies in human prostate cell lines suggested that ETV4 shares with other ETS proteins a major role in invasiveness [30–32] and in cell migration [33, 34]. We have previously found that, unlike other ETS proteins [8–10], ETV4 increases the rate of proliferation of prostate cells and accelerates the progression through the cell cycle [34].

Cyclin-dependent kinases inhibitors (CDKIs) are negative regulators of cell cycle progression. Specifically, p21/CIP1 (encoded by *CDKN1A* gene) and p27/KIP1 (encoded by *CDKN1bB* gene) [35, 36] belong to the Cip/Kip family of CDKIs proteins, and they regulate the progression from quiescence to G1 and from G1 to S phase by inhibiting the activity of the cyclin/CDK complexes [37, 38]. p21 and p27 have been regarded as tumor-suppressor genes and their loss has been associated with poor prognosis in several solid tumors [39–43] including prostate cancer [44–47]. However, the prognostic significance of these proteins in prostate cancer is still controversial [48, 49], especially with respect to p21.

Overall, clinical evidence [25, 50] and in vitro studies [33, 34] strongly suggest that ETV4 plays a key role in prostate cancer in a non-negligible proportion of patients. However, the role of ETV4 overexpression in prostate cancer has never been investigated in vivo.

Here, we report a novel transgenic mouse model in which the overexpression of human ETV4 in the prostate results in late development of mouse prostatic intraepithelial neoplasia (mPIN). In these ETV4-overexpressing mice, we found an increased cell proliferation rate associated with the downregulation of p21 and p27. We further show that ETV4 downregulation of p21 (*CDKN1A*) is determined not only through direct binding of ETV4 to the *CDKN1A* promoter but also through the downregulation of the p53 protein.

## Materials and methods

### Generation and genotyping of transgenic mice

**Fig. 1:**
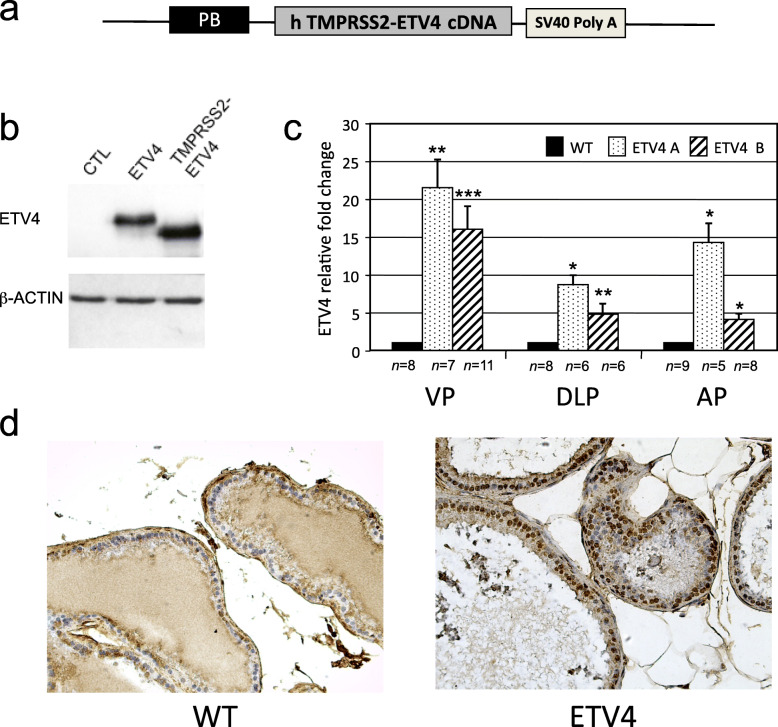
h*ETV4* transgene is expressed in prostate of two transgenic mice lines with prostate-specific ETV4 expression. **a** Diagram of the pPB-ETV4 construct used to generate transgenic mice. It contains part of the rat probasin PB promoter (PB), the *TMPRSS2-ETV4* fusion cDNA, and the SV40 polyadenylation sequence. **b** Western blot analysis of Hek 293 cells transiently transfected with an empty vector (CTL), with the expression vectors containing either the full-length ETV4 (ETV4) or the *TMPRSS2-ETV4* fusion cDNA (TMPRSS2-ETV4) under the control of the ubiquitous EF1a promoter. Beta actin was used as loading control. **c** Relative expression levels of *ETV4* mRNA measured by qRT-PCR in the prostate lobes (VP, DLP, AP) of ETV4 transgenic mice (lines ETV4 A and ETV4 B) normalized to wild-type mice (WT). The data represent mean and standard error of triplicate analysis in the indicated number of mice (*n*). VP: ventral prostate; DLP: dorso-lateral prostate; AP: anterior prostate. *P ≤ 0.05; **P ≤ 0.01; ***P ≤ 0.001. **d** Representative microphotographs of ETV4 protein expression by immunohistochemistry analysis (magnification × 200) in the prostate of WT mice (left panel) and ETV4 mice (right panel)

The rat probasin promoter (PB) was excised from the ARR2PBCAT plasmid [51] and cloned within the previously described TME vector [34] to generate the pPB-ETV4 vector that express the *TMPRSS2-ETV4* fusion cDNA under the control of PB promoter (Fig. 1a). A 3.96 kb fragment—containing PB, *TMPRSS2-ETV4*, and SV40 polyA sequences—was excised from pPB-ETV4 vector (Fig. 1a) and used for pronuclear injections into FVB mouse fertilized eggs that were implanted into pseudo-pregnant females at the LiGeMA Facility of the University of Florence, Italy. Potential founder animals were screened by PCR using primers specific for human *ETV4* (hETV4) and the murine *β-actin* (Table S1), and characterized for construct integration by southern blot analyses (Fig. S1). This animal study was performed in compliance with relevant regulatory standards, and it was approved by the Institutional Animal Care and Use of University of Florence, Italy, and Italian Ministry of Health.

**Table 1:** Number of FVB mice carrying mPIN lesions at different age

Age	6 months	10–11 months
**Histology**	mPIN*	mPIN*
wt	0/10	0/13
ETV4 (line A)	0/5	13^§^/18 (1.6 ± 0.2)
ETV4 (line B)	0/3	4/11 (1.5 ± 0.3)

*The numbers of mice with mPIN relative to the number of mice analyzed is shown. The average number ± sem of mPIN per affected mouse is reported within parentheses

^§^Four mice have both mPIN1 and mPIN2 and 2 mice have only mPIN2

### Cell culture and transfection

Hek-293T, PC3, (IST “Cell Bank and Cell Factory,” Genoa, Italy), PNT1A, and RWPE (American Type Culture Collection) cell lines were cultured according to cell-bank instructions and transfected as previously described [34].

### Histopathologic analysis and immunohistochemistry

Murine prostate tissues were fixed in 10% buffered formalin and embedded in paraffin. For histology, sections were stained with hematoxylin and eosin. Pathologic diagnosis was performed following the recommendations of the “Mouse Models of Human Cancer Consortium Prostate Pathology Committee” and the reference classification of mouse prostatic intraepithelial neoplasia (mPIN) in genetically modified animals [52, 53].

For immunohistochemical examination, 4 μm sections were deparaffinized and incubated with the relevant antibodies (Table S2) and visualized using the biotin–streptavidin complex (Thermo Scientific, Rockford, IL, USA) and diaminobenzidine (Dako, Glostrup, Denmark) as chromogen. Slides were then counterstained with hematoxylin. Bromodeoxyuridine (BrdU) incorporation has been evaluated on prostate tissues obtained from 2 transgenic and 2 control mice 12 h after intraperitoneal injection of 100 mg/kg body weight of BrdU and processed as described above. The slides, after antigen retrieval and HCl treatment, were stained with anti BrdU antibody (Abcam, UK).

### Quantitative reverse transcription PCR (qRT-PCR)

RNA extraction and qRT-PCR were performed as previously described [34] with SsoFast EvaGreen Supermix and the CFX96 thermocycler (Bio-Rad Hercules, CA, USA). Expression level of each gene was analyzed by 2ΔΔC(T) method using either murine or human *glyceraldeide 3-phosphate dehydrogenase* as housekeeping gene. The primers are reported in Table S1. Each experiment has been performed at least three times in triplicate.

### Western blot (WB) analysis

Proteins from mouse prostate, after tissue disruption with Tissue lyser II (Qiagen, Germantown, MD, USA), and from cell lines were extracted in RIPA buffer. Western blot analyses were performed as previously described [34] by using antibodies listed in Table S2. Horseradish-peroxidase-conjugated secondary antibody signals were detected using ECL (Pierce, Rockford, IL, USA) and Chemidoc XRS plus (Bio-Rad). Densitometric quantifications have been performed by using the software ImageJ (https://imagej.nih.gov/ij/) [54].

### Chromatin immunoprecitation (ChIP)

RWPE cells were transiently transfected with either pCMV-3Tag-3A empty vector (Clontech, Mountain View, CA, USA) or with its derivative vector, pETV4-3Flag, expressing flagged ETV4. Magna ChIP A/G Chromatin Immunoprecipitation Kit (Mercks-Millipore, Billerica, MA, USA) has been used according to the manufacturer’s instructions. In brief, transfected RWPE cells were fixed with 1% formaldehyde and lysed. DNA was sonicated and diluted with ChIP buffer, and one-tenth of lysate was collected as input control. Chromatin was incubated overnight at 4 °C with G/A magnetic beads pre-conjugated with either anti-Flag antibody (Cell Signaling) or a non-specific IgG control. DNA was purified, and measured by quantitative PCR (qPCR) using the primers reported in Table S1. A fragment of the *COX2* promoter, a well-known ETV4 regulated gene, was used as positive control. A region of *G6PD* gene was used as negative control. ChIP assays were performed at least three times.

### Dual luciferase reporter assays

Dual luciferase reporter experiments were performed using Dual-Glo Luciferase Assay System and the GloMax 20/20 Luminometer (Promega, Madison, WI, USA). Each firefly luciferase pGL4.20 reporter vector (listed below) was used in combination with renilla luciferase pRL-TK reporter vector (Promega) (ratio 10:1) to normalize luciferase activity. The relative luciferase activity (Firefly/Renilla ratio) was measured in (i) RWPE cells transfected with an ETV4-expressing vector (FL-ETV4) [34] normalized to those transfected with an empty vector, and in (ii) PC3 cells transfected with vectors containing shRNA against ETV4 and normalized to those transfected with a scrambled shRNA (siRNA anti-ETV4 and scrambled siRNA—Dharmacon, Lafayette, CO, USA—have been used in some experiments). Each experiment has been performed at least three times in quadruplicate.

A set of luciferase pGL4.20 reporter vectors, in which luciferase is driven by putative ETV4 responsive elements from the human *CDKN1A* promoter (GenBank Accession #NC_000006.12), were generated as follows. The 845-bp (from 36677905 to 36678749) and the 1656-bp (from 36677084 to 36678739) fragments were amplified using the primers listed in Table S1. These fragments, containing putative ETV4-binding sites, were cloned within the luciferase pGL4.20 reporter vector to obtain the ETV4 responsive luciferase vectors ETV4-BS-A and ETV4-BS-AB, respectively. Derivative vectors were obtained mutating the putative ETV4-binding sites with the QuikChange II site-Directed Mutagenesis Kit (Agilent Technologies, Santa Clara, CA, USA): the normal sequence “CCGGAAGC” of the ETV4 BS A (Figs. 5a, c and 6a) was replaced with the mutated sequence “CCGATATC”; the normal sequence “AGAGGAAGAA” of ETV4 BS-B (Figs. 5a and 6a) was replaced with the mutated sequence “AACCGAAGAA.” In addition, also a p53-responsive luciferase reporter vector (Addgene) [55] was used.

### Statistical analysis

All data are expressed as mean ± sem. Student’s *t* test or one-way ANOVA (followed by Bonferroni correction), as suitable, were performed using GraphPad Prism v.5.0 for Windows (GraphPad Software, La Jolla, CA, USA). Statistical significance was accepted for *P* ≤ 0.05.

## Results

### Generation of mice expressing hETV4 in the prostate

In the fusion transcript *TMPRSS2-ETV4*, found in prostate cancer patients, a sequence upstream of the *TMPRSS2* gene is juxtaposed to the last 9 bp of intron 2 of *ETV4* gene [25]. This results in a protein lacking the first 39 amino acids of normal ETV4, as the start codon becomes an ATG in *ETV4* exon 4. To assess in vivo the pathogenic role of ETV4 in prostate, we engineered a vector that expresses this ETV4-encoded shortened protein [34] under the control of a modified rat probasin promoter (PB) (Fig. 1a, b) that is able to drive an androgen-inducible prostate-specific expression [56]. By using this PB-ETV4 vector, we have obtained six founders in the FVB strain: three of these were able to transmit the transgene to their progeny and two of them expressed the exogenous hETV4 in the prostate of the derived mouse lines (ETV4-A, ETV4-B) (Fig. 1c, d). Very low levels of hETV4 were detected in the seminal vesicles but not in other tissues. The levels of murine etv4 were not affected by the expression of the hETV4 (data not shown). By southern blot analysis of founders and offspring we determined that in every mouse of these two lines the transgene was present at a single integration site (Fig. S1): this ensures uniform genetic transmission of the transgene.

The expression of the h*ETV4* transgene was different in the distinct lobes that constitute the mouse prostate with the highest levels in the ventral lobe. Indeed, the increase in total ETV4 expression in our transgenic mice compared with wild-type mice was 18.6 ± 2.4 in the ventral prostate (VP, *P* < 0.0001); 5.9 ± 1.3 in the dorso-lateral prostate (DLP, *P* < 0.009) and 7.4 ± 1.9 in the anterior prostate (AP, *P* < 0.03). ETV4 expression was increased in both transgenic mouse lines but it was higher in the ETV4-A line (Fig. 1c), the difference between the two lines was statistically significant only in the AP (*P* < 0.001).

### Transgenic expression of hETV4 in the mouse prostate induces prostatic intraepithelial neoplasia (PIN)

**Fig. 2:**
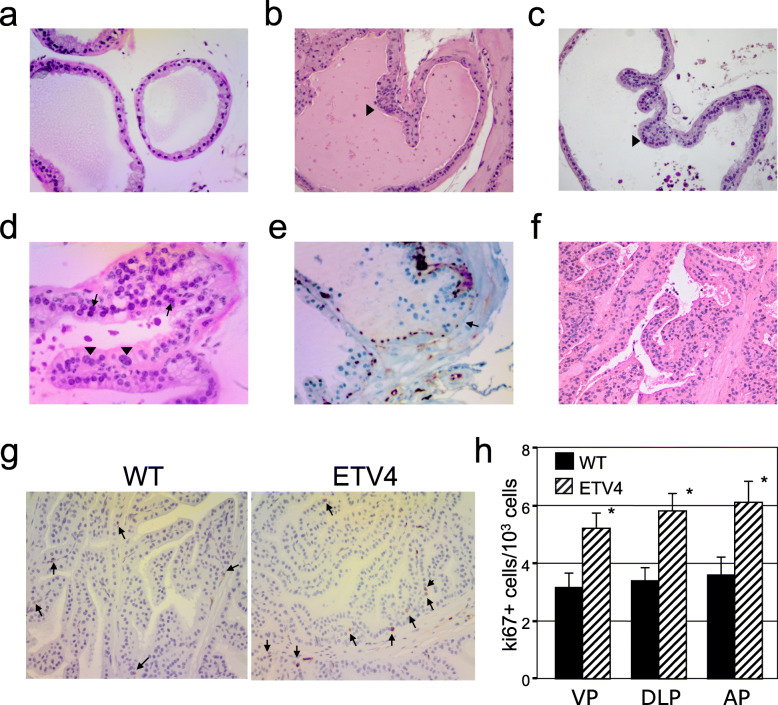
ETV4 expression induces prostatic intraepithelial neoplasia in old mice and increases cell proliferation in prostate. Prostate of wild-type (WT) mice (**a**) and ETV4 transgenic mice from lines A (**b**) and B (**c**) at 11 months (hematoxylin and eosin, magnification × 200). Mouse prostatic intraepithelial neoplasia (mPIN) are indicated by arrowheads in **b** and **c**. A minority of cells displayed macronuclei (arrowhead in **d**) or prominent nucleoli (arrows in **d**). mPIN foci showed loss of p63-positive basal cells (arrows in **e**). In rare instances, epithelial cells have cribriform or tufting patterns and exhibited increasingly severe nuclear pleomorphism and hyperchromasia (**f**). **g** Cell proliferation analysis by Ki67 staining in the anterior prostate of 5-month-old wild-type (left panel) and ETV4 mice (right panel). Arrows indicate proliferating cells. **h** Bar diagram of the frequency of Ki67-positive cells in the 3 prostate lobes from 7 mice. Average and standard error values are shown. *P ≤ 0.05

Neither gross nor microscopic prostate lesions were found in FVB wild-type (*n* = 10) or transgenic mice (*n* = 8) before 6 months of age. At 10–11 months, focal atypical lesions of the prostate epithelium were seen in 13 out of 18 (72%) transgenic mice from line A and in 4 out of 11 (36%) transgenic mice from line B (Table 1). These lesions were characterized by crowding and stratification of luminal cells (Fig. 2b, c) with variable degrees of nuclear atypia in the form of nuclear enlargement, pleomorphism, and hyperchromasia (Fig. 2d); they were identified as mouse prostatic intraepithelial neoplasia (mPIN). Immunohistochemical analysis showed loss of p63-positive basal cells, a major diagnostic criterion of PIN in humans (Fig. 2e). The average number of these lesions was 1.6 ± 0.2 in line A and 1.5 ± 0.3 in line B. The majority of lesions were classified as mPIN1; however, six mice from line A also showed the more severe phenotype of mPIN2 (Fig. 2f; Table 1). There was no evidence of invasive growth.

### ETV4 modulates matrix metalloproteinases (MMPs) in vivo and in vitro

ETV4 has an important role in cell motility and in the invasiveness of prostate, breast, and colon cancer cells through the regulation of matrix metalloproteinases (MMPs) that cause degradation of extracellular matrix [30, 57]. We have previously shown in two human prostate cellular models (cancerous PC3 and normal RWPE cell lines) that ETV4 expression regulates migration in the wound-healing assay and invasion in Matrigel through the transcriptional regulation of some MMPs [34]. In ETV4 mice (*n =* 7), we found no variations of *MMP*s expression in AP; however, we found increased expression of *MMP2*, *MMP7*, and *MMP9* mRNA in VP (3.0 ± 0.7, 3.7 ± 0.7, and 2.5 ± 0.7 fold respectively; *P* < 0.05) and in DLP (8.6 ± 3.5; 4.7 ± 1.8, and 1.8 ± 0.3 folds respectively; *P* < 0.08 not significant), suggesting that ETV4 regulates MMPs expression also in vivo.

### ETV4 affects cell proliferation by modulating of *Cdkn1a* and *Cdkn1b* in vivo and in vitro

**Fig. 3.**
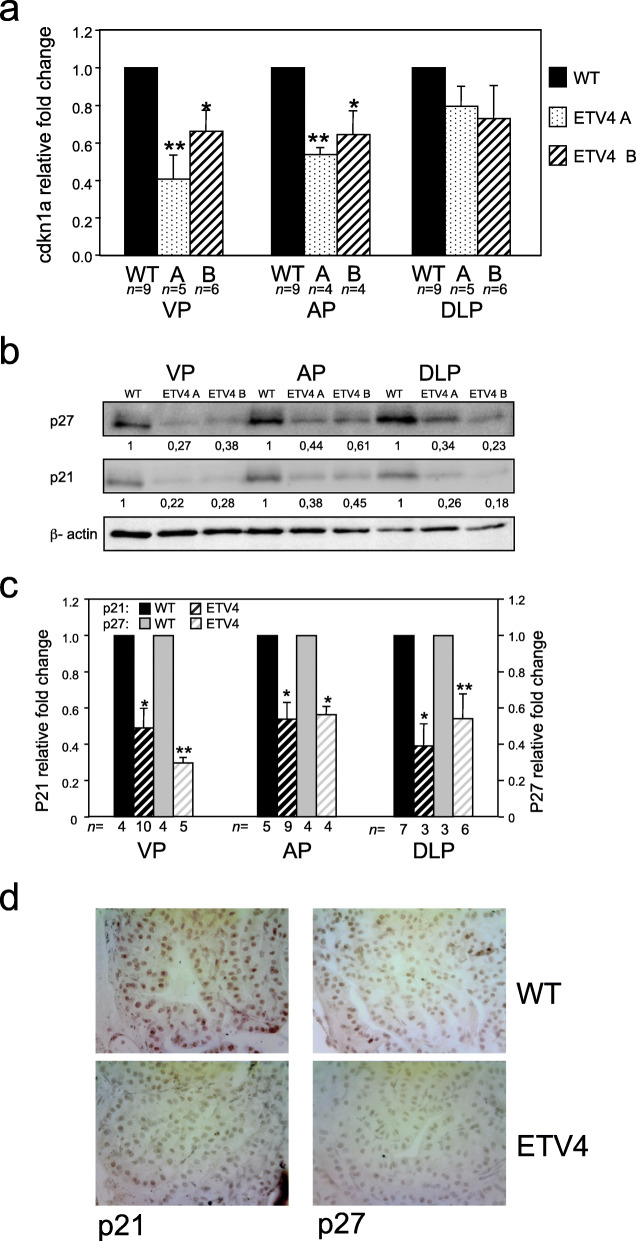
Cell proliferation induced by ETV4 is due to the modulation of p21 and p27. **a** Relative expression levels of murine *Cdkn1a* mRNA measured by qRT-PCR in the prostate lobes (VP, DLP, and AP) of ETV4 transgenic mice (lines ETV4 A and ETV4 B) normalized to wild-type mice (WT). The analysis has been performed in triplicate in the indicated number of mice (*n*). Average and standard error values are shown. *P ≤ 0.05, **P ≤ 0.01. **b** Representative western blots of p21 and p27 proteins in the 3 prostate lobes of wild-type (WT) and of ETV4 transgenic mice (lines ETV4 A and ETV4 B); beta-actin was used as loading control. The relative densitometric quantifications are shown for each lane. **c** Bar diagram of murine p21 and p27 protein levels measured by western blot analysis in the prostate lobes (VP, DLP, and AP) of ETV4 transgenic mice (ETV4) normalized to wild-type mice (WT). Average and standard error values are shown; *n*: number of mice. *P ≤ 0.05, **P ≤ 0.01. **d** Representative microphotograps of p21 (on the left) and p27 (on the right) expression showed by immunohistochemistry analysis in the anterior prostate of WT and ETV4 mice (magnification × 200)

The role of ETV4 in proliferation has not been broadly studied; however, we have previously demonstrated in human prostate cell lines that ETV4 overexpression have a role in cell proliferation [34], and this effect has been associated with the modulation of a set of cell cycle-regulating genes. In order to verify whether ETV4 increases the proliferation rate also in vivo, we measured the percentage of the Ki67+ cells in the prostate (Fig. 2g). In 5-month-old ETV4 mice, the percentage of proliferating (Ki67+) prostate cells was significantly increased in comparison with wild-type mice (*P* < 0.05; Fig. 2h), and this was confirmed also by PCNA staining (Fig. S2a,b) and BrdU incorporation (Fig. S2c, d). Thus, we analyzed two cell-cycle regulatory proteins of Cip/Kip family of cdk inhibitors, the cyclin-dependent kinase inhibitor 1A (*Cdkn1a*) and cyclin-dependent kinase inhibitor 1B (*Cdkn1b*) [58] in the prostate of ETV4 mice. We found a reduced expression of *Cdkn1a* mRNA in both ETV4 mouse lines compared with wild-type mice (Fig. 3a): the reduction was statistically significant in VP (*P* < 0.001) and AP (*P* < 0.01), but not yet in DLP (because the large amount of non-prostatic tissues in DLP or, alternatively, because prostatic lobes may differ in the gene expression pattern). No variation of *Cdkn1b* mRNA level was observed in ETV4 mice (data not shown). At variance, western blot (Fig. 3b, c) and immunohistochemical staining (Fig. 3d) showed that both p21 (encoded by *Cdkn1a*) and p27 (encoded by *Cdkn1b*) proteins were reduced.

**Fig. 4:**
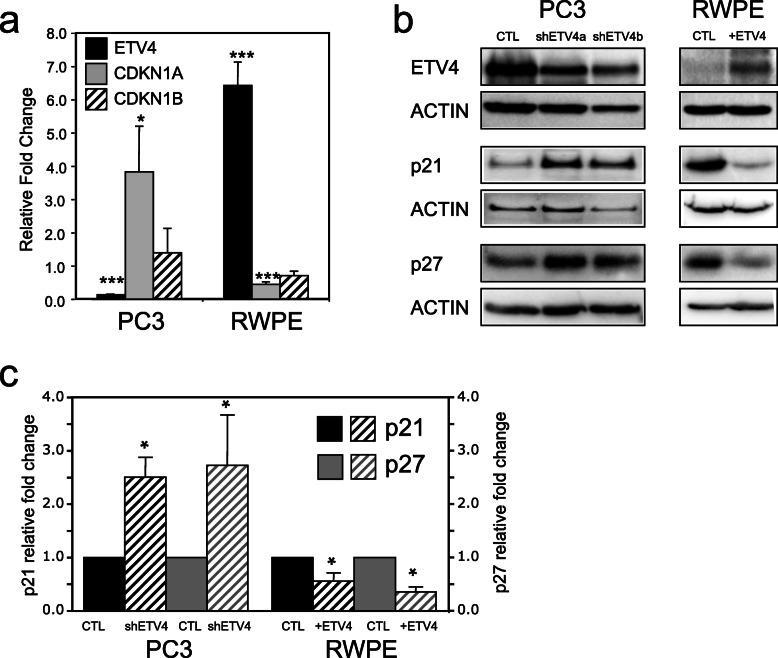
ETV4 regulate p21 (CIP1/WAF1) and p27 (KIP1) expression in human prostate cell lines. **a** Relative expression levels of *CDKN1A*, *CDKN1B*, and *ETV4* mRNA measured by qRT-PCR. PC3 cells were transduced with either an anti-ETV4 shRNA or an irrelevant shRNA. RWPE cells were transfected with either an ETV4-expressing vector or an empty vector. The expression changes are indicated as the ratio between the levels of a given transcript in the experiment and the relevant control. Average and standard error values are shown. At least 4 independent experiments have been performed. *P ≤ 0.05, ***P ≤ 0.001. **b** Representative western blots of p21 and p27 proteins in PC3 cells transduced with either an irrelevant shRNA (CTL) or 2 anti-ETV4 shRNA (shETV4a and shETV4b), and in RWPE cells transfected with either an empty vector (CTL) or an ETV4-expressing vector (+ETV4). Beta-actin is used as loading control. **c** Bar diagram of p21 and p27 protein levels measured by western blot analysis in (i) PC3 cells transduced with either a specific shRNA (shETV4) or an irrelevant shRNA (CTL), and in (ii) RWPE cells transfected with either an empty vector (CTL) or an ETV4-expressing vector (+ETV4). Average and standard error values of the relative quantification are shown. At least 4 independent experiments have been performed. *P ≤ 0.05

In order to confirm that ETV4 regulates negatively *CDKN1A* and *CDKN1B* also in human prostate cells, we have tested the expression levels of these genes and of their encoded proteins (p21/WAF1/CIP1 and p27/KIP1, respectively) in two cellular models: in PC3 cells in which the high endogenous level of ETV4 expression was reduced by specific shRNAs and in RWPE cells in which we overexpressed ETV4. In keeping with the in vivo data and confirming our previous observations, we found that in both these specular cellular models ETV4 modulated the expression of p21 at both mRNA and protein level (Fig. 4), whereas p27 expression was modulated at protein level (Fig. 4b, c) but only slightly and non-significantly at mRNA level (Fig. 4a).

PC3 cells, in addition to ETV4, express also low amounts of ETV1 [59]. ETV4 silencing does not significantly affect ETV1 levels and vice versa (Fig. S3). In addition, ETV1 silencing does not significantly affect p21 and p27 levels (Fig. S3). Thus, the co-expression of ETV4 and ETV1 in this experimental setting is not complementary and does not invalidate our results.

### ETV4 modulates *CDKN1A* levels through direct interaction with its promoter

**Fig. 5:**
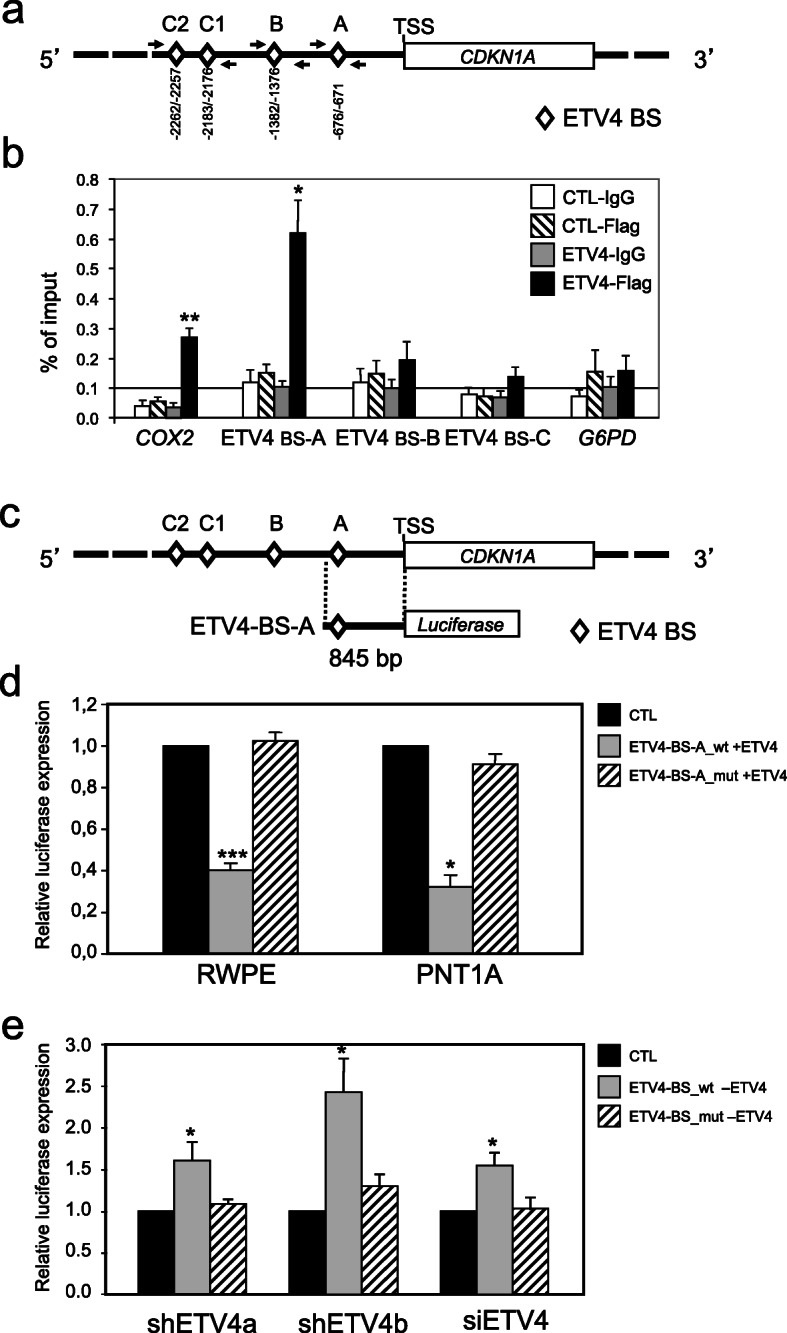
ETV4 binds and downregulates the *CDKN1A* promoter. **a** Putative ETV4-binding sites (ETV4 BS) on *CDKN1A* promoter are indicated by a diamond. The numbers indicate the position of ETV4 BS relative to the transcription start site (TSS). The localization of the primers for the qPCR is indicated. **b** qPCR analysis of ChIP performed on RWPE cells transiently transfected with either the ETV4-Flag expressing vector (ETV4) or a vector expressing only Flag (CTL), the chromatin was immunoprecipitated using the anti-flag antibody (CTL-Flag and ETV4-Flag) or IgG (CTL-IgG and ETV4-IgG) as controls. The signals obtained from the ChIP are expressed as percentage of the imput sample. ETV4-binding sites (ETV4 BS-A, BS-B, BS-C) as shown in **a**. Positive control: *COX2* ETV4-binding site. Negative controls: a fragment from *G6PD* gene. **c** Diagram of the human *CDKN1A* promoter (top) and of the luciferase vector (ETV4-BS-A) containing the 845 bp region upstream the *CDKN1A* TSS (bottom). Diamonds indicate ETV4 BS. **d** Quantification of dual luciferase reporter assay in RWPE and PNT1A cells transiently transfected with vectors in which firefly luciferase expression is driven by the 865 bp *CDKN1A* promoter fragment (see above) containing either the wild-type (ETV4-BS-A_wt) or the mutant (ETV4-BS-A_mut) ETV4 BS-A. The bar diagram shows the relative luciferase activity (Firefly/Renilla ratio) from cells transfected with an ETV4-expressing vector (+ETV4, grey, or striped bars) normalized to those transfected with an empty vector (CTL, black bars). Data represent average and standard error of triplicate measurements of at least 3 independent experiments. **e** Quantification of dual luciferase reporter assay in PC3 cells transiently transfected with luciferase vectors containing either the wild-type (ETV4-BS-A_wt) or the mutant (ETV4-BS-A_mut) ETV4 BS-A (see above). The bar diagram shows the relative luciferase activity (Firefly/Renilla ratio) from cells with ETV4 silencing (–ETV4, grey, or striped bars) normalized to those without ETV4 silencing (CTL, black bars). ETV4 silencing has been obtained with either one of 2 ETV4-specific shRNA (shETV4a, shETV4b) or a siRNA against ETV4 (siETV4). Data represent average and standard error of triplicate measurements of at least 4 (shETV4) or 3 (siETV4) independent experiments. *P ≤ 0.05, **P ≤ 0.01, ***P ≤ 0.001

To determine whether *CDKN1A* expression was regulated directly by ETV4, we studied the binding of ETV4 to the promoter of human *CDKN1A* by chromatin immunoprecipitation (ChIP) assays. First, by using the TESS-Transcription Element Search System software (http://www.cbil.upenn.edu/tess) and the Tfsitescan/dynamicPlus server (http://www.ifti.org/cgi-bin/ifti/Tfsitescan.pl) (Fig. 5a), we identified several ETV4-binding sites (BSs) in a 2.5 kb region upstream (sequence NG_009364 from GenBank) to the transcriptional start site (TSS).

Next, we performed ChIP in RWPE cells transiently transfected with either an ETV4-Flag expressing vector or a Flag-only control vector. The lysates from transfected cells were immunoprecipitated with either anti-flag antibody or an IgG control and the precipitated DNA fragments were PCR-amplified by using a pair of primers complementary to sequences of the *CDKN1A* promoter surrounding the ETV4 BSs (Table S1, Fig. 5a). As positive control, we used the sequence containing the ETV4 BS in the promoter of *COX2* [60], a gene that is directly regulated by ETV4. As negative control, we used a sequence in the *G6PD* gene. The quantitative PCR (q-PCR) results suggested that ETV4 binds to only one of the putative BSs in *CDKN1A* promoter: the proximal ETV4 BS A (BS-A) at -676/-671 from TSS (Fig. 5b).

The effect of the binding of ETV4 to the BS-A site was analyzed by dual luciferase reporter assay, using a vector in which the firefly luciferase is under the control of a 845 bp fragment of the *CDKN1A* promoter that includes the ETV4 BS-A (Fig. 5c). In both RWPE and PNT1A (two immortalized non-cancer human prostate cell lines), the overexpression of ETV4 reduced the luciferase expression from the *CDKN1A* promoter (Fig. 5d). This effect was abrogated when a mutation was introduced into BS-A (Fig. 5d). As a counter-proof of the effects of ETV4 overexpression, we tested a human cancer prostate cell line, PC3, that has high basal levels of ETV4. In these cells, ETV4 silencing by vectors expressing ETV4-specific shRNA (shETV4a or shETV4b) increased the luciferase expression driven by the *CDKN1A* promoter (Fig. 5e). Again, no variation in luciferase expression was observed when the ETV4 BS-A was mutated (Fig. 5e). These results were confirmed also when ETV4 expression was silenced by a specific siRNA against ETV4 (Fig. 5e). These mirror experiments (ETV4 overexpression in PNT1A and RWPE cells; ETV4 silencing in PC3 cells) concur in suggesting that ETV4 negatively regulates *CDKN1A* by interacting with the proximal BS-A in the promoter of this gene.

**Fig. 6:**
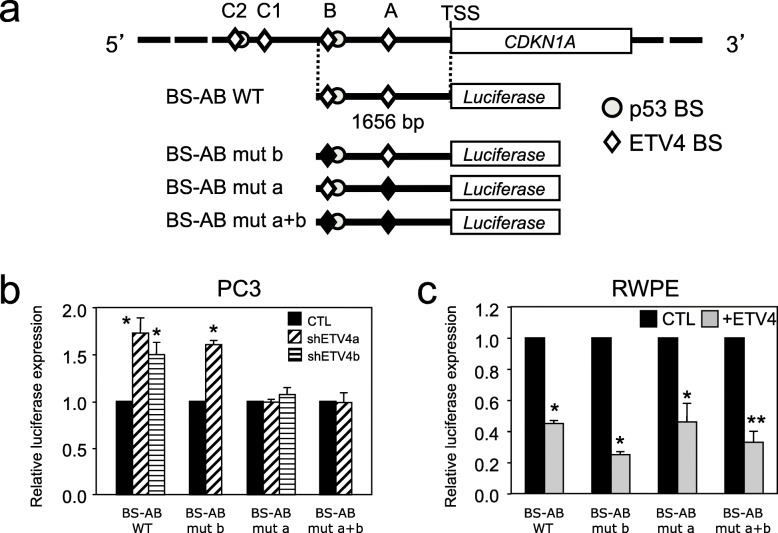
ETV4 downregulates *CDKN1A* promoter regardless of ETV4-binding sites. **a** Diagram of the human *CDKN1A* promoter (top) and of the vectors (BS-AB) in which firefly luciferase expression is driven by the 1656 bp region upstream the *CDKN1A* TSS (bottom). ETV4 BSs are indicated by a diamond and p53 BSs are indicated by a circle. Four different variants of the BS-AB vector have been used. The BS-AB WT with both wild-type ETV4 BS-A and BS-B. In the other 3 vectors, one or both of this ETV4 BS have been mutated; the mutated sites are in black the normal sites are in white. **b** Quantification of dual luciferase reporter assay in PC3 cells transiently transfected with the indicated four BS-AB vectors (see above). The bar diagram shows the relative luciferase activity (Firefly/Renilla ratio) from cells transduced with one of 2 anti-ETV4 shRNA (shETV4a and shETV4b) normalized to those transduced with an irrelevant shRNA (CTL). Data represent average and standard error of triplicate measurement of 3 independent experiments. **c** Quantification of dual luciferase reporter assay in RWPE cells transiently transfected with the indicated four BS-AB vectors (see above). The bar diagram shows the relative luciferase activity (Firefly/Renilla ratio) from cells transfected with an ETV4-expressing vector (+ETV4) normalized to those transfected with an empty vector (CTL). Data represent average and standard error of triplicate measurement of 3 independent experiments. *P ≤ 0.05, **P ≤ 0.01

In order to further confirm this conclusion, we performed dual luciferase reporter assay in PC3 and RWPE cells transfected with a vector containing the firefly luciferase under the control of a larger section of the *CDKN1A* promoter, a 1656 bp fragment that includes the ETV4 BS-A and the putative ETV4 BS-B that had not been confirmed by ChIP (Fig. 6a). Again, we also used derivatives of this vector in which one or both of these ETV4 BSs had been mutated (Fig. 6a). As expected, in PC3 cells, the reduction of ETV4 expression by two shRNA resulted in the increase of luciferase expression, compared with control cells, only with the wild-type ETV4 BS-A but not when it was mutated (Fig. 6b). In RWPE cells, the overexpression of ETV4 reduced luciferase expression with wild-type ETV4 BS-A but, surprisingly, also with the mutated ETV4 BS-A (Fig. 6c).

**Fig. 7:**
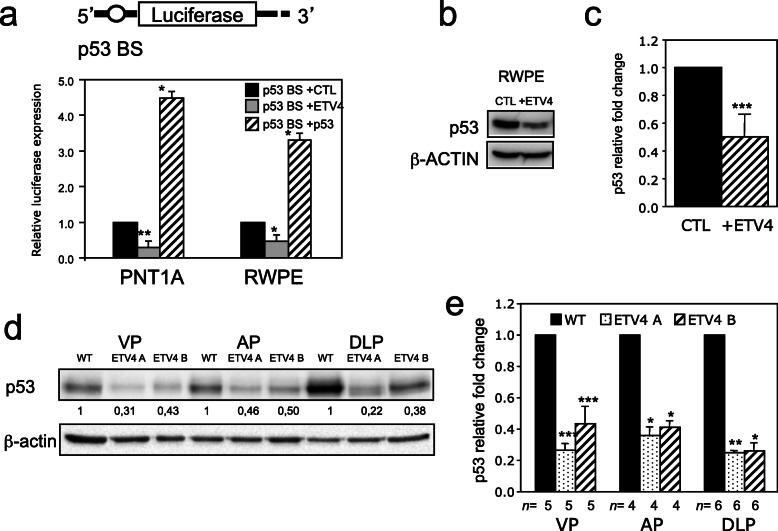
ETV4 downregulates the *CDKN1A* promoter also through the modulation of p53 levels. **a** Dual luciferase reporter assay in PNT1A and RWPE cells transiently transfected with a vector containing firefly luciferase downstream the 20 bp human *CDKN1A* p53-binding site (p53 BS) (top panel). The bar diagram shows the relative luciferase activity (Firefly/Renilla ratio) from cells transfected with either an ETV4-expressing vector (+ETV4, grey bars) or a p53-expressing vector (+p53, striped bars), and normalized to those transfected with an empty vector (CTL, black bar). Data represent average and standard error of triplicate measurements of 3 independent experiments. **b** Representative western blots of p53 in human RWPE cells transfected with either an empty vector (CTL) or an ETV4-expressing vector (+ETV4). Beta actin is used as loading control. **c** Bar diagram of p53 protein levels measured by western blot analysis in human RWPE cells transfected with an ETV4-expressing vector (+ETV4) normalized to those transfected with an empty vector (CTL). Average and standard error values of the relative quantification are shown. Seven independent experiments have been performed. **d** Representative western blots of p53 protein in the 3 prostate lobes of wild-type (WT) and of ETV4 transgenic mice (lines ETV4 A and ETV4 B); beta-actin was used as loading control. The relative densitometric quantifications are shown for each lane. **e** Bar diagram of p53 protein levels measured by western blot analysis in the 3 prostate lobes of ETV4 transgenic mice (lines ETV4 A and ETV4 B) normalized to wild-type mice (WT). Average and standard error values of the relative quantification are shown; *n*: number of mice. *P ≤ 0.05; **P ≤ 0.01; ***P ≤ 0.001

This last unexpected result raised the possibility that the 1656 bp fragment of the *CDKN1A* promoter might contain one or more elements responsive to a gene potentially regulated by ETV4 rather than ETV4 itself. Since the PC3 cells are p53 null, while the RWPE cells are p53 competent, p53 itself seemed like a good candidate because p53 is known to regulate p21 and a p53 BS is present in the *CDKN1A* promoter near to the ETV4 BS-B [55]. By luciferase assay with a vector containing only the p53 biding site (Fig. 7a) of the *CDKN1A* promoter [55], we found in the PNT1A and RWPE cell lines that transient overexpression of ETV4 reduced the luciferase expression compared to control cells (Fig. 7a).

Since these experiments suggest that ETV4 might regulate p53 expression, we tested the effect of ETV4 expression on p53 levels. In human RWPE, prostate cell line ETV4 overexpression does not modify the levels of p53 mRNA (data not shown); however, the levels of p53 protein are reduced (Fig. 7b, c). Although we do not yet know the mechanism, the same is true in the prostate of ETV4 mice (Fig. 7d, e).

## Discussion

Aberrant overexpression of an ETS protein in the prostate is a common event in most of the patients with prostate cancer. This overexpression, caused by the translocation of an *ETS* gene under the control of the promoter of a gene highly expressed in the prostate, plays a direct role in prostate cancer pathogenesis [2, 4]. The most frequent translocation is that of *ERG* gene downstream the promoter of *TMPRSS2*, but rearrangements of *ETV1*, *ETV5*, and *ETV4* genes are also relatively common [25]. The role of ERG and ETV1 in prostate cancer has been thoroughly studied, whereas the mechanisms whereby overexpression of ETV4 mediates oncogenesis in the prostate have not been investigated in depth.

Overexpression of ETV4 confers several neoplastic features onto prostate cell lines [33, 34]; here, we have investigated whether this holds true also in vivo. To this end, we have generated two independent lines of transgenic mice in which the prostate specific expression of *ETV4* is driven by the probasin promoter (ETV4 mice). ETV4 mice showed an increased prostate expression of *MMP2*, *MMP7*, and *MMP9* suggesting that also in vivo ETV4 exerts a transcriptional upregulation of MMPs as observed in human prostate cell lines [34] where it is associated with increased migration and invasion [33, 34].

Two-thirds of 10-month-old ETV4 mice developed prostate focal lesions resembling the early modifications observed in human PIN: in some of these mice some lesions have more severe features. Thus, ETV4 overexpression promotes mPIN with long latency and partial penetrance. However, even in the older mice (15 months) and despite of MMPs overexpression, mPIN did not progress to prostate cancer, implying that additional genetic events are required. These findings are very similar to those reported in various model of ERG and ETV1 prostate transgenic mice [8–10, 12].

The role of ETS proteins, such as ERG and ETV1, in regulating prostate cell proliferation in vivo is minimal: in fact, in ERG and ETV1 transgenic mice, there is no [10, 13, 14] or only a slight increase [8, 9, 12, 16–18] in cell proliferation. However, in a mouse model obtained by pronuclear injection of a bacterial artificial chromosome carrying the *TMPRSS2-ERG* transgene, ERG drives proliferation and blocks the differentiation of prostate cells [61]. In ETV4 mice, instead, we find that ETV4 plays a role in prostate cell proliferation in vivo. This is supported by the significant increase of proliferating cells observed in the prostate of ETV4 mice. This result is consistent with in vitro studies in which ETV4 increases proliferation of human prostate cell lines through progression of cell cycle [34], although cell cycle progression has not yet been proven in ETV4 mice.

In prostate cell lines (RWPE and PC3), ETV4 expression is associated with an increased rate of proliferation and with downregulation of *CDKN1A* [34]. Accordingly, also in ETV4 mice, the increased proliferation rate is associated with reduced levels of both *CDKN1A* mRNA and of its encoded protein p21 (Fig. 3). In addition, also in vivo ETV4 expression is associated with the reduction of p27 protein, but not of its mRNA (encoded by *CDKN1B* gene) as previously found in RWPE cells transfected with ETV4 [34]. ETV4 may promote cell proliferation also in other systems where it regulates a number of genes: *HER2* in ovarian (SKOV-3) and breast (MDA-MB-453) cancer cell lines [62]; *WT1* in CHO and COS7 cell lines [63]; *cyclin D3* in MDA-MB-231 breast cancer cell line [42]; *cyclin D1* in mammary tissues [64]; *NOTCH1* and *NOTCH4* in MCF-7, MDA-MB-231, and SKBr3 breast cancer cells [65]. Taken together, these data suggest that ETV4 can control cell proliferation through a variety of cell type specific mechanisms.

In the prostate, ETV4 hinders the transcription of *CDKN1A* and therefore the level of p21 through direct binding to a proximal ETV4-binding site within the *CDKN1A* promoter (Fig. 5). This mechanism of downregulation of *CDKN1A* in human prostate (RWPE, PNT1A, and PC3) cell lines is reminiscent of the downregulation induced by ETV4 through binding to other promoters, such as that of *ERBB2* promoter in breast and ovarian cancer cells [62, 66] and that of collagenase-1 promoter in a breast cell line treated with all-trans retinoic acid [67]. On the other hand, at variance with p21 (*CDKN1A*), the ETV4-mediated reduction of the protein level of p27 (*CDKN1B*), another cell cycle inhibitor controlling the progression at G1, seems indirect because it is not associated with significant variation of mRNA levels.

In the 1990s, ETV4 was regarded as a tumor suppressor gene because it was able to increase luciferase expression driven by the *CDKN1A* promoter in SiHA cervical cancer cells whereas its deletion reduced *CDKN1A* levels in Saos2 osteosarcoma cells [68]. However, we find that ETV4 downregulates *CDKN1A* in prostate cells and also in MCF7 breast cancer cell line (Fig. S4). These observations suggest that the role of ETV4 in cancer is cell-type and tissue-dependent; it behaves as an oncogene in prostate and breast cells (and in many others tissues) [69], whereas it acts as a tumor suppressor gene in osteosarcoma [68] and in cervical cells [62]. This apparent discrepancy could be explained by the fact that different cell types express different tissue-specific factors that, in turn, may influence the role of ETV4 as it happens for others ETS transcription factors [5, 70].

Direct downregulation of the *CDKN1A* promoter by ETV4 is only part of the story. In fact, ETV4 is able to reduce *CDKN1A* expression even when its binding site to the *CDKN1A* promoter is mutated (Fig. 6). Since this takes place only in p53 competent cells and even when the promoter contains only the p53-binding site [55] led us to realize that regulation of *CDKN1A* by ETV4 is mediated in part through p53. In keeping with this notion, the levels of p53 protein are reduced upon expression of ETV4 in normal human prostatic RWPE cells (Fig. 7b, c), and also in vivo in ETV4 mice (Fig. 7d, e). As already observed for p27, this ETV4-mediated reduction of p53 protein was not associated with any change in *TP53* mRNA, suggesting an indirect regulatory mechanism that remains to be identified.

Our main finding is that both in vitro and in vivo ETV4 can modulate cell cycle and, in turn, proliferation of prostate cells through multiple layers of regulation including both the direct regulation of transcription of some gene (*CDKN1A*-p21) and the indirect regulation of other genes (*CDKN1B*-p27, *TP53*). Finally, the finding that ETV4 reduces the level of p53 protein suggests its possible contribution to cellular processes, beyond cell cycle, in which p53 plays a role, such as apoptosis, genomic stability, and senescence.

ETV4 overexpression in the prostate is observed in only a relatively small subset of prostate cancer patients (about 1–5%) [2–4, 29, 50]; however, since prostate cancer is common, these results are potentially relevant for a significant number of patients. In addition, Aytes and colleagues have reported the late increase of ETV4 expression in a prostate metastasis mouse model with several genetic alterations (pTen loss, NKX3.1 deletion, and a KRAS activating mutation) suggesting that ETV4 may have a role in the metastatic process even in prostate cancers that do not overexpress ETV4 initially [71]. It is also noteworthy that in a recent study of biopsy cores from 120 patients ETV4 expression was mostly associated with high-grade cancer [29]. Thus, the relevance of our results could be extended to an even larger number of patients.

## Conclusions

ETV4 overexpression increases the proliferation rate of prostate cells in vitro and in vivo through both direct and p53-mediated downregulation of *CDKN1A* and its p21 protein product. This may explains the development of mPIN in ETV4 mice. However, mPIN develops after a long latency period and does not progress to cancer, implying that additional genetic events are required. These ETV4 transgenic mice could help to identify new downstream oncogenic pathways and could be used for preclinical testing of drugs in vivo.

## Supplementary information

**Additional file 1: Supplementary Table 1.** Sequences of primers and oligonucleotides. **Supplementary Table 2.** Primary antibodies used in this study. **Supplementary Figure 1.** Southern blot analysis of transgene insertion in ETV4 mouse lines. **Supplementary Figure 2.** Analysis of prostate cell proliferation in mice. **Supplementary Figure 3.** ETV1 expression in PC3 cells. **Supplementary Figure 4.** ETV4 binds and downregulates the *CDKN1A* promoter also in MCF7 breast cancer cell line.

## Data Availability

All data generated and analyzed during this study are included in this published article and its additional file.

## Abbreviations

AP: Anterior prostate
BS: Binding site
CDK: Cyclin-dependent kinases
CDKI: Cyclin-dependent kinases inhibitors
CDKN1A: Cyclin-dependent kinase inhibitor 1A
CDKN1B: Cyclin-dependent kinase inhibitor 1B
ChIP: Chromatin immunoprecipitation assay
COX2: Cyclooxygenase 2
CTL: Control
DLP: Dorso-lateral prostate
EF1a: Elongation factor 1A
ERG: ETS-related gene
ETV1: ETS variant gene 1
ETV4: ETS variant gene 4
FL: Full length
GAPDH: Glyceraldehyde-3-phosphate dehydrogenase
G6PD: Glucose 6 phosphate dehydrogenase
MMP: Metalloproteinase
mPIN: Mouse prostatic intraepithelial neoplasia
PB: Rat probasin promoter
PC: Prostate cancer
qPCR: Quantitative PCR
qRT-PCR: Quantitative reverse transcription PCR
SV40: Simian virus 40
TMPRSS2: Transmembrane serine protease 2
TSS: Transcription start site
VP: Ventral prostate
WB: Western blot
WT: Wild-type

## References

[1] Siegel RL, Miller KD, Jemal A (2019). Cancer statistics, 2019. CA Cancer J Clin..

[2] Rubin MA, Maher CA, Chinnaiyan AM (2011). Common gene rearrangements in prostate cancer. J Clin Oncol..

[3] Paulo P, Barros-Silva JD, Ribeiro FR, Ramalho-Carvalho J, Jeronimo C, Henrique R, et al. FLI1 is a novel ETS transcription factor involved in gene fusions in prostate cancer. Genes Chromosomes Cancer. 2012;51(3):240–9. 10.1002/gcc.20948 22081504

[4] Arora K, Barbieri CE (2018). Molecular subtypes of prostate cancer. Curr Oncol Rep..

[5] Nicholas TR, Strittmatter BG, Hollenhorst PC (2019). Oncogenic ETS factors in prostate cancer. Adv Exp Med Biol..

[6] Yu C, Hu K, Nguyen D, Wang ZA (2019). From genomics to functions: preclinical mouse models for understanding oncogenic pathways in prostate cancer. Am J Cancer Res..

[7] Arriaga JM, Abate-Shen C (2019). Genetically engineered mouse models of prostate cancer in the postgenomic era. Cold Spring Harb Perspect Med..

[8] Tomlins SA, Laxman B, Dhanasekaran SM, Helgeson BE, Cao X, Morris DS (2007). Distinct classes of chromosomal rearrangements create oncogenic ETS gene fusions in prostate cancer. Nature..

[9] Klezovitch O, Risk M, Coleman I, Lucas JM, Null M, True LD (2008). A causal role for ERG in neoplastic transformation of prostate epithelium. Proc Natl Acad Sci U S A..

[10] Tomlins SA, Laxman B, Varambally S, Cao X, Yu J, Helgeson BE (2008). Role of the TMPRSS2-ERG gene fusion in prostate cancer. Neoplasia..

[11] Zong Y, Xin L, Goldstein AS, Lawson DA, Teitell MA, Witte ON (2009). ETS family transcription factors collaborate with alternative signaling pathways to induce carcinoma from adult murine prostate cells. Proc Natl Acad Sci U S A..

[12] Shin S, Kim TD, Jin F, van Deursen JM, Dehm SM, Tindall DJ (2009). Induction of prostatic intraepithelial neoplasia and modulation of androgen receptor by ETS variant 1/ETS-related protein 81. Cancer Res..

[13] Carver BS, Tran J, Chen Z, Carracedo-Perez A, Alimonti A, Nardella C (2009). ETS rearrangements and prostate cancer initiation. Nature.

[14] Carver BS, Tran J, Gopalan A, Chen Z, Shaikh S, Carracedo A (2009). Aberrant ERG expression cooperates with loss of PTEN to promote cancer progression in the prostate. Nat Genet..

[15] King JC, Xu J, Wongvipat J, Hieronymus H, Carver BS, Leung DH (2009). Cooperativity of TMPRSS2-ERG with PI3-kinase pathway activation in prostate oncogenesis. Nat Genet..

[16] Casey OM, Fang L, Hynes PG, Abou-Kheir WG, Martin PL, Tillman HS (2012). TMPRSS2- driven ERG expression in vivo increases self-renewal and maintains expression in a castration resistant subpopulation. PLoS One..

[17] Baena E, Shao Z, Linn DE, Glass K, Hamblen MJ, Fujiwara Y (2013). ETV1 directs androgen metabolism and confers aggressive prostate cancer in targeted mice and patients. Genes Dev..

[18] Chen Y, Chi P, Rockowitz S, Iaquinta PJ, Shamu T, Shukla S (2013). ETS factors reprogram the androgen receptor cistrome and prime prostate tumorigenesis in response to PTEN loss. Nat Med..

[19] Hida K, Shindoh M, Yoshida K, Kudoh A, Furaoka K, Kohgo T (1997). Expression of E1AF, an ets-family transcription factor, is correlated with the invasive phenotype of oral squamous cell carcinoma. Oral Oncol..

[20] Benz CC, O'Hagan RC, Richter B, Scott GK, Chang CH, Xiong X (1997). HER2/Neu and the Ets transcription activator PEA3 are coordinately upregulated in human breast cancer. Oncogene..

[21] de Launoit Y, Chotteau-Lelievre A, Beaudoin C, Coutte L, Netzer S, Brenner C (2000). The PEA3 group of ETS-related transcription factors. Role in breast cancer metastasis. Adv Exp Med Biol..

[22] Hiroumi H, Dosaka-Akita H, Yoshida K, Shindoh M, Ohbuchi T, Fujinaga K (2001). Expression of E1AF/PEA3, an Ets-related transcription factor in human non-small-cell lung cancers: its relevance in cell motility and invasion. Int J Cancer..

[23] Moss AC, Lawlor G, Murray D, Tighe D, Madden SF, Mulligan AM (2006). ETV4 and Myeov knockdown impairs colon cancer cell line proliferation and invasion. Biochem Biophys Res Commun..

[24] Upadhyay S, Liu C, Chatterjee A, Hoque MO, Kim MS, Engles J (2006). LKB1/STK11 suppresses cyclooxygenase-2 induction and cellular invasion through PEA3 in lung cancer. Cancer Res..

[25] Tomlins SA, Mehra R, Rhodes DR, Smith LR, Roulston D, Helgeson BE (2006). TMPRSS2:ETV4 gene fusions define a third molecular subtype of prostate cancer. Cancer Res..

[26] Han B, Mehra R, Dhanasekaran SM, Yu J, Menon A, Lonigro RJ (2008). A fluorescence in situ hybridization screen for E26 transformation-specific aberrations: identification of DDX5-ETV4 fusion protein in prostate cancer. Cancer Res..

[27] Hermans KG, Bressers AA, van der Korput HA, Dits NF, Jenster G, Trapman J (2008). Two unique novel prostate-specific and androgen-regulated fusion partners of ETV4 in prostate cancer. Cancer Res..

[28] Iljin K, Wolf M, Edgren H, Gupta S, Kilpinen S, Skotheim RI (2006). TMPRSS2 fusions with oncogenic ETS factors in prostate cancer involve unbalanced genomic rearrangements and are associated with HDAC1 and epigenetic reprogramming. Cancer Res..

[29] Dedigama-Arachchige P, Carskadon S, Li J, Loveless I, Alhamar M, Peabody JO*,* et al. Clonal evaluation of prostate cancer molecular heterogeneity in biopsy samples by dual immunohistochemistry and dual RNA in situ hybridization. Mod Pathol. 2020. Online ahead of print. 10.1038/s41379-020-0525-0 32238875

[30] Higashino F, Yoshida K, Noumi T, Seiki M, Fujinaga K (1995). Ets-related protein E1A-F can activate three different matrix metalloproteinase gene promoters. Oncogene..

[31] Horiuchi S, Yamamoto H, Min Y, Adachi Y, Itoh F, Imai K (2003). Association of ets-related transcriptional factor E1AF expression with tumour progression and overexpression of MMP-1 and matrilysin in human colorectal cancer. J Pathol..

[32] Shindoh M, Higashino F, Kohgo T (2004). E1AF, an ets-oncogene family transcription factor. Cancer Lett..

[33] Hollenhorst PC, Paul L, Ferris MW, Graves BJ (2011). The ETS gene ETV4 is required for anchorage-independent growth and a cell proliferation gene expression program in PC3 prostate cells. Genes Cancer..

[34] Pellecchia A, Pescucci C, De Lorenzo E, Luceri C, Passaro N, Sica M (2012). Overexpression of ETV4 is oncogenic in prostate cells through promotion of both cell proliferation and epithelial to mesenchymal transition. Oncogenesis..

[35] Grana X, Reddy EP (1995). Cell cycle control in mammalian cells: role of cyclins, cyclin dependent kinases (CDKs), growth suppressor genes and cyclin-dependent kinase inhibitors (CKIs). Oncogene..

[36] Chu IM, Hengst L, Slingerland JM (2008). The Cdk inhibitor p27 in human cancer: prognostic potential and relevance to anticancer therapy. Nat Rev Cancer..

[37] Cheville JC, Lloyd RV, Sebo TJ, Cheng L, Erickson L, Bostwick DG (1998). Expression of p27kip1 in prostatic adenocarcinoma. Mod Pathol..

[38] Drobnjak M, Melamed J, Taneja S, Melzer K, Wieczorek R, Levinson B (2003). Altered expression of p27 and Skp2 proteins in prostate cancer of African-American patients. Clin Cancer Res..

[39] Noda H, Maehara Y, Irie K, Kakeji Y, Yonemura T, Sugimachi K (2001). Growth pattern and expressions of cell cycle regulator proteins p53 and p21WAF1/CIP1 in early gastric carcinoma. Cancer..

[40] Shoji T, Tanaka F, Takata T, Yanagihara K, Otake Y, Hanaoka N (2002). Clinical significance of p21 expression in non-small-cell lung cancer. J Clin Oncol..

[41] Shariat SF, Tokunaga H, Zhou J, Kim J, Ayala GE, Benedict WF (2004). p53, p21, pRB, and p16 expression predict clinical outcome in cystectomy with bladder cancer. J Clin Oncol..

[42] Jiang J, Wei Y, Liu D, Zhou J, Shen J, Chen X (2007). E1AF promotes breast cancer cell cycle progression via upregulation of Cyclin D3 transcription. Biochem Biophys Res Commun..

[43] Somlo G, Chu P, Frankel P, Ye W, Groshen S, Doroshow JH (2008). Molecular profiling including epidermal growth factor receptor and p21 expression in high-risk breast cancer patients as indicators of outcome. Ann Oncol..

[44] Matsushima H, Sasaki T, Goto T, Hosaka Y, Homma Y, Kitamura T (1998). Immunohistochemical study of p21WAF1 and p53 proteins in prostatic cancer and their prognostic significance. Hum Pathol..

[45] Cheng L, Lloyd RV, Weaver AL, Pisansky TM, Cheville JC, Ramnani DM (2000). The cell cycle inhibitors p21WAF1 and p27KIP1 are associated with survival in patients treated by salvage prostatectomy after radiation therapy. Clin Cancer Res..

[46] Graff JR, Konicek BW, McNulty AM, Wang Z, Houck K, Allen S (2000). Increased AKT activity contributes to prostate cancer progression by dramatically accelerating prostate tumor growth and diminishing p27Kip1 expression. J Biol Chem..

[47] Murillo H, Huang H, Schmidt LJ, Smith DI, Tindall DJ (2001). Role of PI3K signaling in survival and progression of LNCaP prostate cancer cells to the androgen refractory state. Endocrinology..

[48] Lacombe L, Maillette A, Meyer F, Veilleux C, Moore L, Fradet Y (2001). Expression of p21 predicts PSA failure in locally advanced prostate cancer treated by prostatectomy. Int J Cancer..

[49] Omar EA, Behlouli H, Chevalier S, Aprikian AG (2001). Relationship of p21(WAF-I) protein expression with prognosis in advanced prostate cancer treated by androgen ablation. Prostate..

[50] Network TCGAR (2015). The Molecular Taxonomy of Primary Prostate Cancer. Cell..

[51] Zhang J, Thomas TZ, Kasper S, Matusik RJ (2000). A small composite probasin promoter confers high levels of prostate-specific gene expression through regulation by androgens and glucocorticoids in vitro and in vivo. Endocrinology..

[52] Shappell SB, Thomas GV, Roberts RL, Herbert R, Ittmann MM, Rubin MA (2004). Prostate pathology of genetically engineered mice: definitions and classification. The consensus report from the Bar Harbor meeting of the Mouse Models of Human Cancer Consortium Prostate Pathology Committee. Cancer Res..

[53] Ittmann M, Huang J, Radaelli E, Martin P, Signoretti S, Sullivan R (2013). Animal models of human prostate cancer: the consensus report of the New York meeting of the Mouse Models of Human Cancers Consortium Prostate Pathology Committee. Cancer Res..

[54] Schneider CA, Rasband WS, Eliceiri KW (2012). NIH Image to ImageJ: 25 years of image analysis. Nat Methods..

[55] el-Deiry WS, Tokino T, Waldman T, Oliner JD, Velculescu VE, Burrell M (1995). Topological control of p21WAF1/CIP1 expression in normal and neoplastic tissues. Cancer Res..

[56] Wu X, Wu J, Huang J, Powell WC, Zhang J, Matusik RJ (2001). Generation of a prostate epithelial cell-specific Cre transgenic mouse model for tissue-specific gene ablation. Mech Dev..

[57] Kaya M, Yoshida K, Higashino F, Mitaka T, Ishii S, Fujinaga K (1996). A single ets-related transcription factor, E1AF, confers invasive phenotype on human cancer cells. Oncogene..

[58] Besson A, Dowdy SF, Roberts JM (2008). CDK inhibitors: cell cycle regulators and beyond. Dev Cell..

[59] Mesquita D, Barros-Silva JD, Santos J, Skotheim RI, Lothe RA, Paulo P (2015). Specific and redundant activities of ETV1 and ETV4 in prostate cancer aggressiveness revealed by co-overexpression cellular contexts. Oncotarget..

[60] Ratovitski EA (2010). LKB1/PEA3/DeltaNp63 pathway regulates PTGS-2 (COX-2) transcription in lung cancer cells upon cigarette smoke exposure. Oxid Med Cell Longev..

[61] Mounir Z, Lin F, Lin VG, Korn JM, Yu Y, Valdez R (2014). TMPRSS2:ERG blocks neuroendocrine and luminal cell differentiation to maintain prostate cancer proliferation. Oncogene..

[62] Xing X, Wang SC, Xia W, Zou Y, Shao R, Kwong KY (2000). The ets protein PEA3 suppresses HER-2/neu overexpression and inhibits tumorigenesis. Nat Med..

[63] Discenza MT, Vaz D, Hassell JA, Pelletier J (2004). Activation of the WT1 tumor suppressor gene promoter by Pea3. FEBS Lett..

[64] Galang CK, Muller WJ, Foos G, Oshima RG, Hauser CA (2004). Changes in the expression of many Ets family transcription factors and of potential target genes in normal mammary tissue and tumors. J Biol Chem..

[65] Clementz AG, Rogowski A, Pandya K, Miele L, Osipo C (2011). NOTCH-1 and NOTCH-4 are novel gene targets of PEA3 in breast cancer: novel therapeutic implications. Breast Cancer Res..

[66] Yu Z, Xia W, Wang HY, Wang SC, Pan Y, Kwong KY (2006). Antitumor activity of an Ets protein, PEA3, in breast cancer cell lines MDA-MB-361DYT2 and BT474M1. Mol Carcinog..

[67] Benbow U, Schoenermark MP, Orndorff KA, Givan AL, Brinckerhoff CE (1999). Human breast cancer cells activate procollagenase-1 and invade type I collagen: invasion is inhibited by all-trans retinoic acid. Clin Exp Metastasis..

[68] Funaoka K, Shindoh M, Yoshida K, Hanzawa M, Hida K, Nishikata S (1997). Activation of the p21(Waf1/Cip1) promoter by the ets oncogene family transcription factor E1AF. Biochem Biophys Res Commun..

[69] Oh S, Shin S, Janknecht R (2012). ETV1, 4 and 5: an oncogenic subfamily of ETS transcription factors. Biochim Biophys Acta..

[70] Li R, Pei H, Watson DK (2000). Regulation of Ets function by protein - protein interactions. Oncogene..

[71] Aytes A, Mitrofanova A, Kinkade CW, Lefebvre C, Lei M, Phelan V (2013). ETV4 promotes metastasis in response to activation of PI3-kinase and Ras signaling in a mouse model of advanced prostate cancer. Proc Natl Acad Sci U S A..

