# Supplementary material for: ETV4 promotes late development of prostatic intraepithelial neoplasia and cell proliferation through direct and p53-mediated downregulation of p21
Source: J Hematol Oncol. 2020 Aug 13;13:112. doi: 10.1186/s13045-020-00943-w (PMC7427297; doi:10.1186/s13045-020-00943-w)
Supplement: Supplementary file 1 — Additional file 1: Supplementary Table 1. Sequences of primers and oligonucleotides. Supplementary Table 2. Primary antibodies used in this study. Supplementary Figure 1. Southern blot analysis of transgene insertion in ETV4 mouse lines. Supplementary Figure 2. Analysis of prostate cell proliferation in mice. Supplementary Figure 3. ETV1 expression in PC3 cells. Supplementary Figure 4. ETV4 binds and downregulates the CDKN1A promoter also in MCF7 breast cancer cell line. [file 13045_2020_943_MOESM1_ESM.pdf]

Supplementary tables and figures

Title:

ETV4 promotes late development of prostatic intraepithelial neoplasia and cell proliferation through direct and p53-mediated downregulation of p21

Authors

Irene Cosi[1]*, Annamaria Pellecchia[1]*, Emanuele De Lorenzo[1]*, Eugenio Torre[2], Michela Sica[1], Gabriella Nesi[3], Rosario Notaro[1], Maria De Angioletti[1,4].

Supplementary Table 1: Sequences of primers and oligonucleotides.

Region	Procedure	Forward primer sequence	Reverse primer sequence
human ETV4	genotyping	5'-CTGGACATTTGCCACTCCTT-3'	5'-AAAGCTCTGCTGGGGATAGG-3'
murine beta-actin	genotyping	5′- ACCACAGCTGAGAGGGAAATC -3	5′- AGAGGTCTTTACGGATGTCAA -3
human GAPDH	qRT-PCR	5'- AACGGATTTGGTCGTATTGGGC-3'	5'- TTGATTTTGGAGGGATCTCG -3'
murine gapdh	qRT-PCR	5'- TGACCACAGTCCATGCCATC-3'	5'- GACGGACACATTGGGGGTAG -3'
* human and murine ETV4	qRT-PCR	5'-ACCACCAGGATCAAGAAGGA-3'	5'-GGCACTGGAGTAAAGGCACT-3'
human ETV4	qRT-PCR	5'-CCACCAGGATCAAGAAGGAG-3'	5'-CTCAGGAAATTCCGTTGCTC-3'
murine etv4	qRT-PCR	5'-GGCAGAACAGCAGCAGAGC-3'	5'-CCTCCCTGAGGAGATGTGAA-3'
murine cdkn1a	qRT-PCR	5'-GCAGATCCACAGCGATATCC-3'	5'-CAACTGCTCACTGTCCACGG-3'
murine cdkn1b	qRT-PCR	5'-AAGGGCCAACAGAACAGAAG-3'	5'-GGATGTCCATTCAATGGAGTC-3'
murine tp53	qRT-PCR	5'-AACCGCCGACCTATCCTTAC-3'	5'-CTTCTGTACGGCGGTCTCTC-3'
human CDKN1A	qRT-PCR	5'-CTAGGCGGTTGAATGAGAGG-3'	5'-GCCAGGGTATGTACATGAGGA-3'
human CDKN1B	qRT-PCR	5'-AATAAGGAAGCGACCTGCAA-3'	5'-TTCTGAGGCCAGGCTTCTT-3'
human TP53	qRT-PCR	5'-AACCGCCGACCTATCCTTAC-3'	5'-CTTCTGTACGGCGGTCTCTC-3'
murine mmp2	qRT-PCR	5'-CAAGTTCCCCGGCGATGTC-3'	5'-TTCTGGTCAAGGTCACCTGTC-3'
murine mmp7	qRT-PCR	5'-GGGGACTGCGGATATCATAA-3'	5'-AAAGTGAGCATCTCCGCCTA-3'
murine mmp9	qRT-PCR	5'-GTGGTTCAGTTGTGGTGGTG-3'	5'-CCCGCTGTATAGCTACCTCG-3'
human *COX2*	ChIP	5'-TCCCTCCTCTCCCCTTAAAA-3'	5'-AGTGGGGACTACCCCCTCTG-3'
human *CDKN1A* promoter (site A)	ChIP	5'-TTCCCTGGAGATCAGGTTGC-3'	5'-TGAGGAAATTGAGGTCCACTG-3'
human *CDKN1A* promoter (site B)	ChIP	5'-CCCCACAGCAGAGGAGAAAG-3'	5'-TGCAGAGGATGGATTGTTCA-3'
human *CDKN1A* promoter (site C)	ChIP	5'-CTGTGGCTCTGATTGGCTTT-3'	5'-CTCCTACCATCCCCTTCCTC-3'
human *G6PD*	ChIP	5'- AGGCCGTGTACACCAAGATGA-3'	5'- CGTAGGCGTCAGGGAGCTT-3'
ETV4-BS (A)-Luc	Cloning	5'-ata**CTCGAG**CTGCAACCACAGGGATTTCT-3' (XhoI)	5'-acg**AAGCTT**CCACAAGGAACTGACTTCG-3' (Hind III)
ETV4-BS (A+B)-Luc	Cloning	5'-CTGTCCTCTG**CTCGAG**ATTTGACAAC-3 (XhoI)	5'-CCAC**AAGCTT**CTGACTTCGGCAG-3'(Hind III)
shETV4A	Silencing	5'-GGCGCTTCCCAACTTCATA-3'	
shETV4B	Silencing	5'-CCCTGTGTACATATAAATGAA-3'	
irrelevant shRNA	Silencing	5'-GCCTATTTACGCCTGACAA-3'	
siRNA ETV4	Silencing	ONTARGETplus SMARTpool ref# SO-2582781G	
irrelevant siRNA	Silencing	ONTARGETplus Non-targeting Pool ref# D-001810	
shETV1	Silencing	5'-TTGTGTTCATACACTGGGTCG-3'	

The procedures in which the primers have been used are reported. * To quantify ETV4 expression in murine prostate we selected primers that recognize ETV4 sequences identical in mouse and human. To improve cloning efficiency, the respective primers include sequences (in **bold**) of sites for restriction enzyme indicated in parenthesis.

Supplementary Table 2. Primary antibodies used in this study.

Antigen	clone	Procedure	Producer	Catalog number
ß-actin	AC-15	WB	Sigma-Aldrich, St Louis, MO, USA	A1978
ETV4	3G9-1B9	WB	Abnova, Atlanta, GA, USA	H00002118-M01
ETV4	polyclonal	IHC	Life Span Bioscience, Seattle, WA, USA	LS-B1527
Ki67	B56	IHC	Pharmigen, San Diego, CA, USA	550609
p63	4A4	IHC	NeoMarkers, Fremont, CA, USA	M1081-P1
p21 Waf1/Cip1	C19	WB	SantaCruz Biotechnologies, Dallas TX, USA	sc397G
p21 Waf1/Cip1	2F1	IHC/WB in mice	Abnova	H00001026-M02
p27 Kip1	polyclonal	WB; IHC	Cell signalling; Boston, MA, USA	2552
p53	DO-1	WB	Santa Cruz Biotechnologies	SC-126
PCNA	poyclonal	IHC	Abcam, Cambridge, UK	ab15497
BrdU	BU1/75 (ICR1)	IHC	Abcam	ab8326

The procedures in which the antibodies have been used are reported.

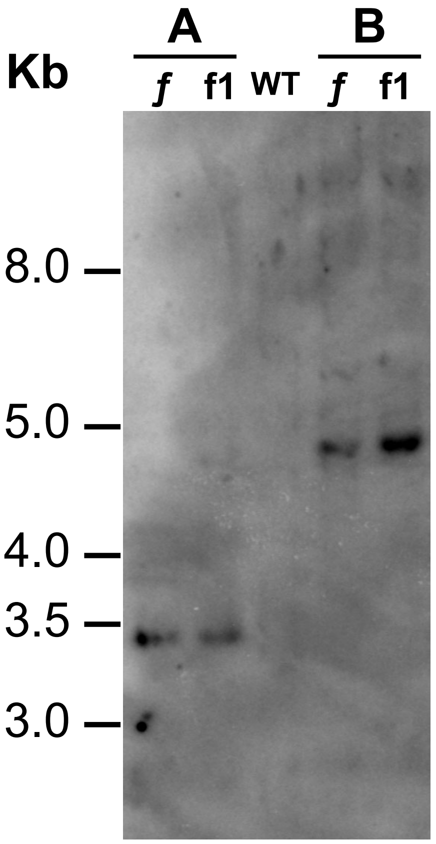

Supplementary Figure 1. Southern blot analysis of transgene insertion in ETV4 mouse lines.

Copy number assessed by Southern blot analysis on DNA from mice belonging to line **A** and line **B**. DNA has been digested by *XbaI*, which recognizes only one site in the PB-ETV4 construct, and probed with the construct. *XbaI* should generate a band larger than 3.0 Kb (the length of the construct) for each different integration site. *ƒ*= founder; f1= F1 progeny; WT= wild-type.

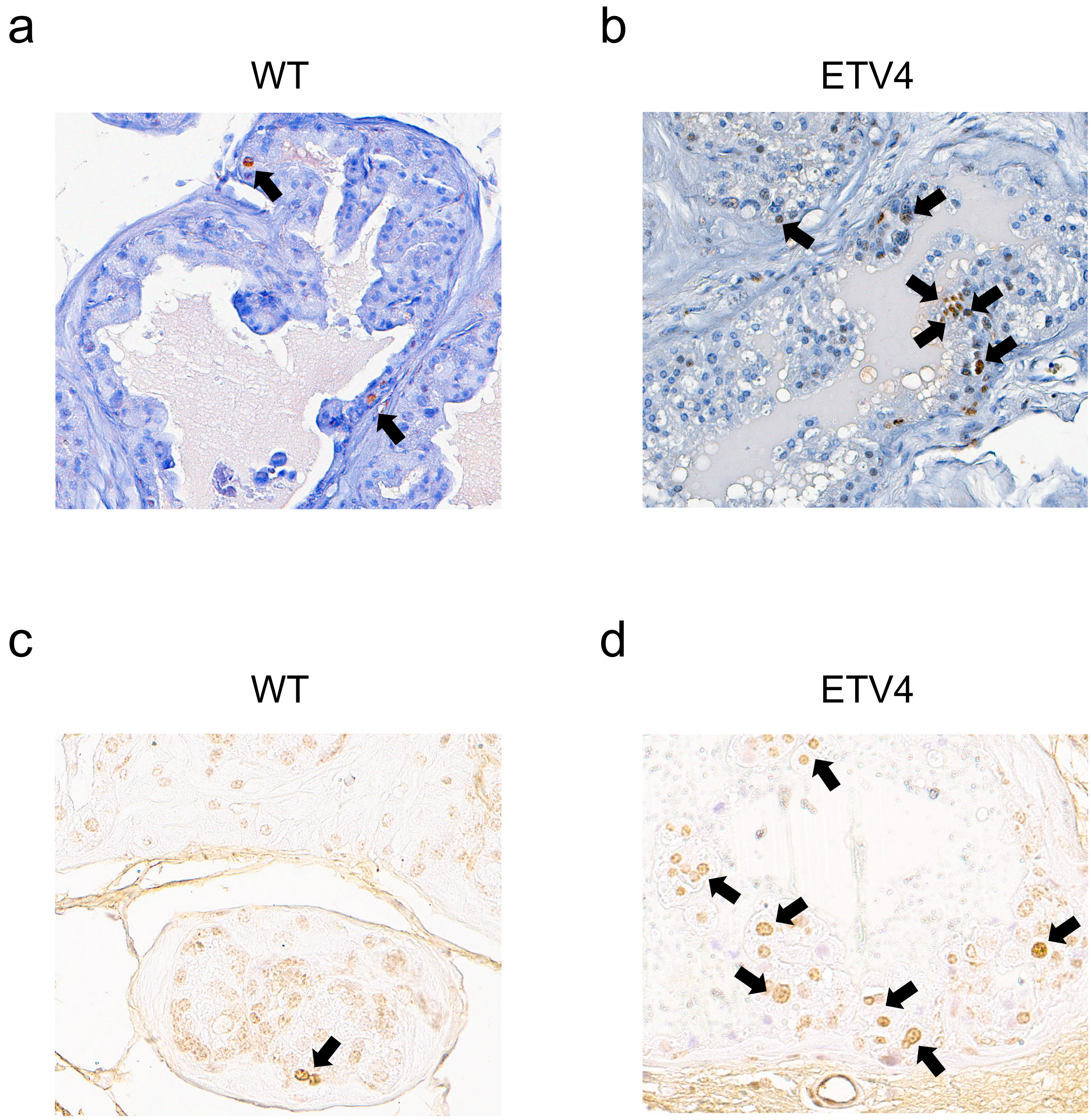

Supplementary Figure 2. Analysis of prostate cell proliferation in mice.

Proliferating cell nuclear antigen (PCNA) staining of mouse prostate cells. Cell proliferation analysis by PCNA staining in the anterior prostate of 5 month-old wild-type (a) and ETV4 mice (b). Arrows indicate proliferating cells.

Bromodeoxyuridine (BrdU) incorporation in mouse prostate cells. Cell proliferation analysis by BrdU incorporation in the anterior prostate of 5 month-old wild-type (c) and ETV4 mice (d). Arrows indicate proliferating cells.

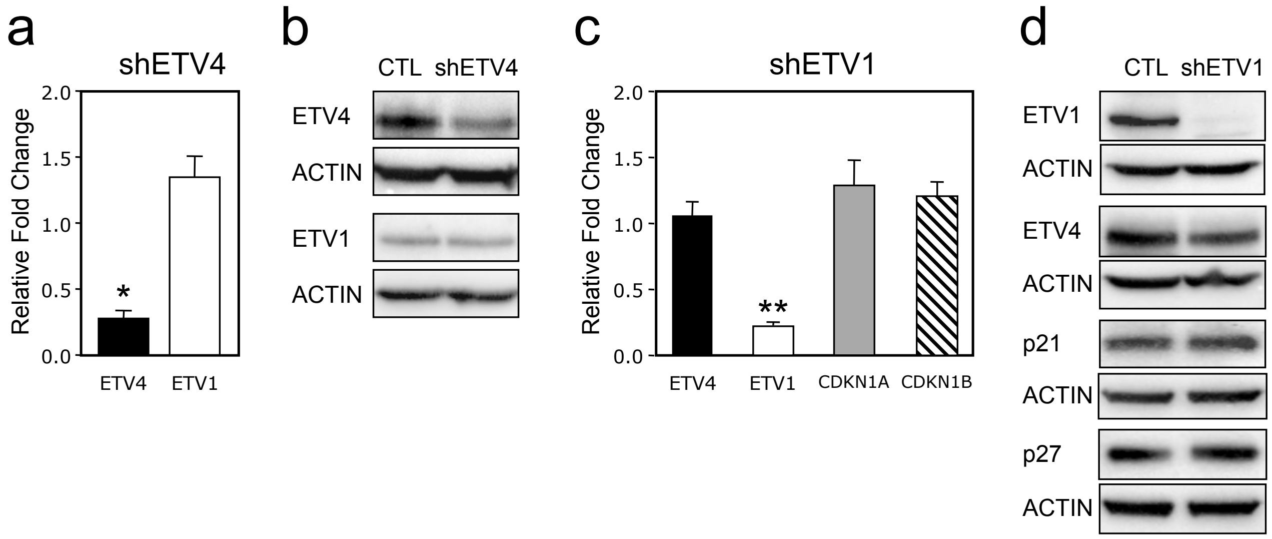

Supplementary Figure 3. ETV1 expression in PC3 cells.

PC3 cells co-express ETV4 and ETV1 (Mesquita *et al.*, *Oncotarget* 2015; 6:5217-36). This co-expression could potentially affect our results. We have investigated the effects of ETV4 silencing on ETV1 expression and the effect of ETV1 silencing on the expression of ETV4, CDKN1A and CDKN1B. These data suggest that the low basal expression of ETV1 in PC3 (about 5%-10% of ETV4 levels: Mesquita *et al.*, *Oncotarget* 2015 and our data) does not interfere with our experimental model. In any event, all our observations have been validated in the mirror model, *i.e.* the RWPE cells transfected with an ETV4-expressing vector.

(a) qRT-PCR of PC3 cells transduced with either an anti-ETV4 shRNA (shETV4) or with an irrelevant shRNA has shown that ETV4 silencing results in a non-significant variation of ETV1 expression. Data represent average and standard error values from 3 independent experiments in triplicate. *$P \leq 0.05$.

(b) Western blots of PC3 cells transduced with either an anti-ETV4 shRNA or with an irrelevant shRNA (CTL) have shown that ETV4 silencing results in a non-significant variation of ETV1 expression. Representative blots of 3 independent experiments.

(c) qRT-PCR of PC3 cells transduced with either an anti-ETV1 shRNA (shETV1) or with an irrelevant shRNA (CTL) has shown that ETV1 silencing results in a non-significant variation of *ETV4*, *CDKN1A and CDKN1B* expression. Data represent average and standard error values from 3 independent experiments in triplicate. **$P \leq 0.01$.

(d) Western blots of PC3 cells transduced with either an anti-ETV1 shRNA (shETV1) or with an irrelevant shRNA (CTL) have shown that ETV1 silencing results in a non-significant variation of ETV4, p21 and p27 expression. Representative blots of 3 independent experiments.

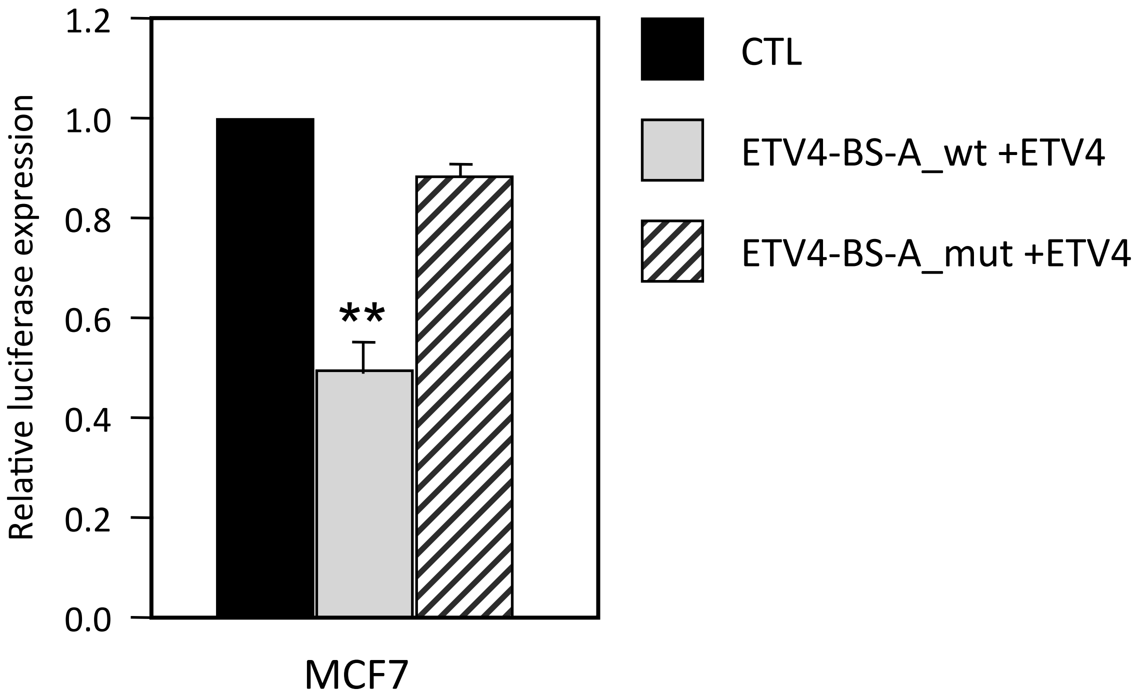

Supplementary Figure 4. ETV4 binds and downregulates the *CDKN1A* promoter also in MCF7 breast cancer cell line.

Quantification of dual luciferase reporter assay in MCF7 breast cancer cell line transiently transfected with vectors in which firefly luciferase expression is driven by the 865 bp CDKN1A promoter fragment (Fig. 5b) containing either the wild-type (ETV4-BS-A_wt) or the mutant (ETV4-BS-A_mut) ETV4 BS-A. The bar diagram shows the relative luciferase activity (Firefly/Renilla ratio) from cells transfected with an ETV4-expressing vector (+ETV4, grey and dashed bars) normalized to those transfected with an empty vector (CTL, black bar). Data represent average and standard error of triplicate measurements of at least 3 independent experiments. ** P≤ 0.01.
